# Prognostic Factors and Nomograms to Predict Overall and Cancer-Specific Survival for Children with Wilms' Tumor

**DOI:** 10.1155/2019/1092769

**Published:** 2019-12-03

**Authors:** Fucai Tang, Hanbin Zhang, Zechao Lu, Jiamin Wang, Chengwu He, Zhaohui He

**Affiliations:** ^1^Department of Urology, The Eighth Affiliated Hospital, Sun Yat-sen University, Shenzhen 518033, China; ^2^The Second Clinical College of Guangzhou Medical University, Guangzhou 511436, China; ^3^The First Clinical College of Guangzhou Medical University, Guangzhou 511436, China; ^4^Department of Urology, Minimally Invasive Surgery Center, Guangdong Provincial Key Laboratory of Urology, The First Affiliated Hospital of Guangzhou Medical University, Guangzhou 510230, China

## Abstract

**Objective:**

This study is aimed at constructing and verifying nomograms that forecast overall survival (OS) and cancer-specific survival (CSS) of children with Wilms' tumor (WT).

**Patients and methods:**

Clinical information of 1613 WT patients who were under 18 years old between 1988 and 2010 was collected from the Surveillance, Epidemiology, and End Results (SEER) database. Using these data, we performed univariate as well as multivariate Cox's regression analyses to determine independent prognostic factors for WT. Then, nomograms to predict 3- and 5-year OS and CSS rates were constructed based on the identified prognostic factors. The nomograms were validated externally and internally. The nomograms' reliability was evaluated utilizing receiver operating characteristic (ROC) curves and concordance indices (C-indices).

**Results:**

1613 WT patients under 18 were involved in the study and randomly divided into the training (*n* = 1210) and validation (*n* = 403) cohorts. Age at diagnosis, tumor laterality, tumor size, tumor stage, and use of surgery were determined as independent prognostic factors for OS and CSS in WT and were further applied to construct prognostic nomograms. The C-index and area under the receiver operating characteristic curve (AUC) revealed the great performance of our nomograms. Internal and external calibration plots also showed excellent agreement between actual survival and nomogram prediction.

**Conclusion:**

Precise and convenient nomograms were developed for forecasting OS and CSS of children with WT. These nomograms were able to offer accurate and individualized prognosis and assisted clinicians in performing suitable therapy.

## 1. Introduction

Wilms' tumor, known as a kind of pediatric cancer, is associated with undifferentiated embryonic lesions. According to the sides of the kidney affected, WT can be classified as unilateral and bilateral tumors. The most common symptom of WT is the presence of an abdominal mass, and hematuria takes second place [1]. It is reported to rank second among the most common tumors of children's abdomen and ranks fifth among the most common pediatric malignancies [2]. Malignant renal tumors account for approximately 6% of the malignancy of children, and 90% of the malignant renal tumors were WT. [3, 4] It is estimated that one child in 10,000 is affected [5]. The incidence of WT is high. Fortunately, the survival rate of children with WT greatly increased due to the development of a therapy in the past several decades. It was reported that the survival rate of WT patients rose from less than 30% in the 1930s to over 90% in 2005 [2], and the annual reduction in risk of death during 1978–2005 was 4% [6].

Individualized therapies depending on their circumstances play a vital role in the treatment process of WT. Precise and individualized therapy not only saves medical resources but is also beneficial to the long-term health of patients. Consequently, it is of significance to determine prognostic factors for individuals with WT as with any malignancies to ensure that the most appropriate therapy is applied to certain individuals. Previous studies which were committed to identifying the prognostic factors for children with WT reported that merely tumor stage and histology had been applied to define therapy until 2015, but clinical practice uses quantities of clinical and biologic factors incorporating age, tumor size and bulk, sensitivity of chemical drug, and loss of heterozygosity (LOH) at chromosomes 1p and 16q [7–10]. Other studies showed that diffuse anaplasia [1], surgery, radiation [11], microscopic residual disease, and lymph node involvement [12] were related with the prognosis of WT. Yet Fernandez et al. [13] proposed that a combination of lymph node and LOH status should be viewed as prognostic factors of stage III favorable-histology WT. Undoubtedly, the prognosis of WT in children is affected by lots of factors simultaneously. To solve this issue, we sought to establish a novel prognostic model.

Nomograms, which are considered as powerful tools, are widely applied to estimate the prognosis of varieties of cancer. Nevertheless, to the best of our knowledge, satisfactory nomograms to predict the survival of children with WT have not been developed. Based on statistical regression models [14], nomograms offer a brand new visible calculating scale method to evaluate the survival rate [15]. Consequently, we aimed to develop effective nomograms to estimate survival rate for children with WT in the present study.

## 2. Patients and Methods

### 2.1. Patients Included and Variables

All the clinical data we needed for the study was obtained from the Surveillance, Epidemiology, and End Results (SEER) database, which incorporated data from eighteen cancer registries [16] and covered almost 34.6 percent of the American public [17]. Patients' baseline features, initial tumor site and stage, primary therapy, and critical follow-up status were available on the website [17], which allowed us to perform comprehensive analyses for these patients. As the clinical data is obtained from the SEER database, it is not necessary to get patients' informed consent and ethics approval due to the absence of case-identifying information [18]. SEER∗Stat software (Version 8.3.5; National Cancer Institute, Bethesda, MD, USA) was applied to obtain patients' information from the database. Patients with complete follow-up were included in this study, and patients' autopsy reports were excluded.

Patients with the following conditions were included: [1] diagnosed with WT as the initial malignancy; [2] aged under 18 years old; [3] histological type confirmation of WT (histologic type ICD-O-3:8960); [4] diagnosed from 1988 to 2010 to guarantee a follow-up time of no less than 3 years; [5] duration between tumor confirmed and death as well as a clear reason for death; and [6] intact follow-up. Patients with the following conditions were excluded: [1] patients without stage, laterality, and surgery information; [2] patients without a definite tumor size, survival time and status, and cancer-specific survival status; and [3] those who were diagnosed at >18 years of age.

**Figure 1:**
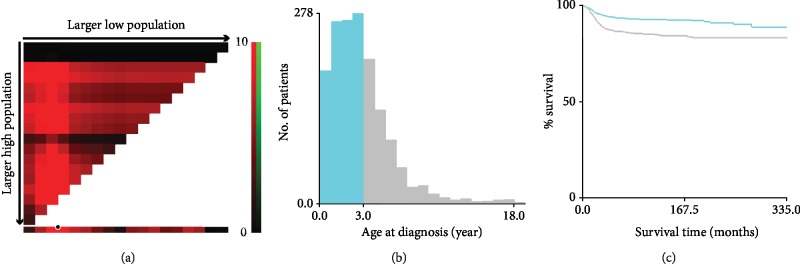
Applying X-tile analysis to determine the best cutoff value of age at diagnosis. (a) The graph shows that the best age cutoff point has been determined by X-tile software. 3.0 years was identified as the optimal cutoff value, and (b) histogram and (c) Kaplan-Meier's analysis were conducted using the optimal cutoff value.

Based on the inclusion and exclusion criteria mentioned above, we initially filtered 1613 patients for the present study. Their vital clinicopathological features including gender, age, race, tumor laterality, tumor size, surgery, SEER historic stage A, and survival time were extracted and further analyzed. The X-tile program (Yale University, New Haven, Connecticut, USA), which was firstly exploited to define the optimal cutoff points of variables for breast cancer patients and has exerted powerful efficacy in defining the optimal cutoff values in other tumors [19],was applied to determine the optimal cut-point of age at diagnosis. The optimal age cutoff was 3.0 years old (Figure 1). Thus, children in the present study were stratified as two groups (0-3 years and 3-18 years). Race included black, white, and others (American Indian, Aleutian, Alaskan Native, or Eskimo). According to the side where the WT originated, tumor laterality was categorized as left, right, and bilateral. We divided patients into three groups which were ≤4 cm, 4-7 cm, and >7 cm by tumor size. Owing to the absence of details of surgery, such as intralesional, wide, or marginal, use of surgery was only classified as yes or no. In terms of the SEER historic stage A, it was categorized as localized, regional, and distant. Since cases “No” and “Unknown” of chemotherapy and radiation were combined as a single option in the updated SEER database, adding this information as a variable might attribute to relevant bias [14, 18]. Hence, our study did not contain these variables.

### 2.2. Statistical Analysis

Children meeting the inclusion standards mentioned above were involved in our study. These children were divided into a training cohort (*n* = 1210) and a validation cohort (*n* = 403) by the random split-sample method (split ratio: 3 : 1). Then, Chi-squared tests were performed to compare the baseline clinicopathological characteristics of the patients in the two cohorts.

We defined overall survival (OS) and cancer-specific survival (CSS) as two primary endpoints of the current study. OS and CSS were defined as the survival time calculated from cancer confirmed to mortality from all probable causes and cancer cause, respectively. Patients who were still alive until the last follow-up were viewed as censored observations.

All the critical variables including gender, diagnosis age, race, tumor laterality, tumor size, use of surgery, and SEER tumor stage A were subjected to univariate Cox's regression analysis of OS and CSS. Variables such as diagnosis age, tumor laterality, tumor size, use of surgery and SEER tumor stage A, which were considered to have statistical significance (*P* < 0.05) in the univariate analyses, were further analyzed with multivariate Cox's regression analysis. All the variables' hazard ratios (HR) and corresponding 95% CI were calculated at the same time.

### 2.3. Establishment and Validation of the Nomograms

Based on the univariate and multivariate Cox's regression analyses, we established nomograms predicting 3- and 5-year OS as well as 3- and 5-year CSS. To assess the nomograms' accuracy, internal and external validations were, respectively, performed in the training and validation cohorts. The area under the receiver operating characteristic curve (AUC) was used for verifying the nomograms. Also, Harrell's concordance index (C-index), which was a powerful tool to appraise nomograms, was utilized to assess the predicting ability of our nomograms. C-indices which ranged from 0.5 to 1.0 and the two points indicated total chance and perfect matching, respectively [18, 20]. Predicted survival and actual outcomes were compared via calibration curves.

SPSS software (version 22.0; IBM Corp., Armonk, NY, USA) was used to perform Chi-squared tests and univariate and multivariate Cox's regression analyses. R package rms was utilized to develop and verify nomograms in R software (version 3.5.3). Results were considered statistically significant on the condition that *P* values were less than 0.05.

## 3. Results

### 3.1. Patient Baseline Characteristics

Overall, 1613 WT patients under 18 years old during 1988-2010 in the SEER database were incorporated in the present study and they were assigned into the training cohort (*n* = 1210) and the validation cohort (*n* = 403) at random. We used the training cohort information for establishing and internally validating the nomograms. And the clinical information of the validation cohort was applied to externally validate the nomograms.

Patients' baseline demographic and clinical characteristics are listed in Table 1. Among the patients, 773 (47.9%) patients were boys and 840 patients (52.1%) were girls. Children under 3 years old had a total number of 1006 (62.4%) and children aged at 3-18 had a total number of 607 (37.6%). In terms of tumor laterality, left had a total number of 754 (46.7%) and right had a total number of 759 (47.1%) nearly accounting for the same proportion, whereas children with bilateral WTs merely accounted for 100 (6.2%). Most tumors (1278 (79.2%)) were larger than 7 cm. 1580 (98.0%) children underwent surgery in the present study. As for SEER historic stage A, localized disease (731 (45.3%)) was the most common, followed by regional disease (503 (31.2%)) and distant metastasis (379 (23.5%)). During the follow-up time, 110 and 16 children died from WT and other causes in the training cohort, respectively. For the validation cohort, 31 and 4 children died from WT and other causes, respectively. Differences for all variables between the training cohort and the validation cohort had no statistical significance (all *P* > 0.05).

### 3.2. Prognostic Factors for OS and CSS

In the training cohort, age at diagnosis, tumor laterality, tumor size, use of surgery, and SEER historic stage A were initially determined to correlate with OS and CSS via univariate Cox's regression analyses (Table 2). For excluding possible confounding factors, multivariate Cox's regression analyses were further performed for these five factors, which indicated that all of these factors (age at diagnosis, tumor laterality, tumor size, use of surgery, and SEER historic stage A) were independent prognostic factors for OS and CSS (Table 3).

### 3.3. Development and Validation of the Prognostic Nomograms

**Figure 2:**
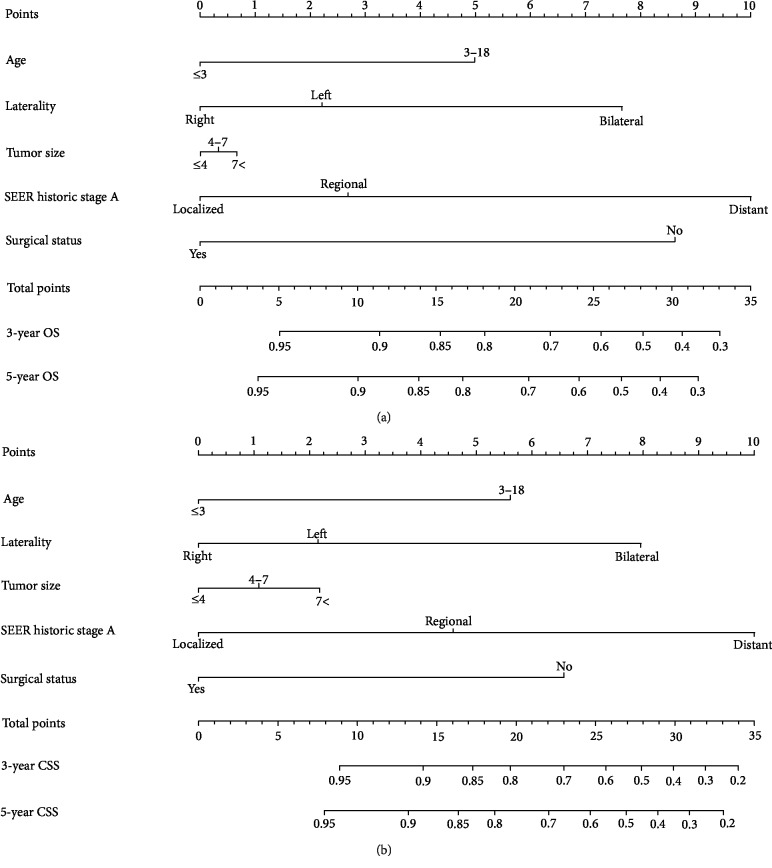
Nomograms predicting 3-year and 5-year overall survival (a) and cancer-specific survival (b) of Wilms' tumor patients. A certain score of each variable can be shown when a perpendicular line between the point scale and each variable is drawn. By adding all the scores as a total score and drawing a perpendicular line between the total point scale and OS or CSS scales, we can estimate the predicted survival rate.

Nomograms were constructed for 3- and 5-year OS and CSS using the independent prognostic factors (Figure 2). All the prognostic factors were given detailed scores based on the analyses (Table 4). With these nomograms, clinicians were able to predict the prognosis of children with WT effortlessly. In these nomograms, the incorporated five variables were age at diagnosis, tumor laterality, tumor size, use of surgery, and tumor stage.

Using these nomograms, we were able to predict 3- and 5-year OS and CSS rates of the children with WT at a negligible cost. According to the individual prognostic factors of children with WT, a detailed score for each variable could be found in Table 4. We could add up these scores and predict the 3- and 5-year OS and CSS. Taking one example, a 10-year-old child was detected with WT as the primary malignancy in his left kidney and the tumor's diameter was 6 cm. Then, no surgery was executed and it was confirmed to be a regional disease. He got 18.8 and 20.0 points for OS and CSS, respectively. In accordance with the nomograms, the corresponding 3-year OS and CSS rates were 78% and 79%, whereas the 5-year OS and CSS were estimated to be 75% and 76%.

**Figure 3:**
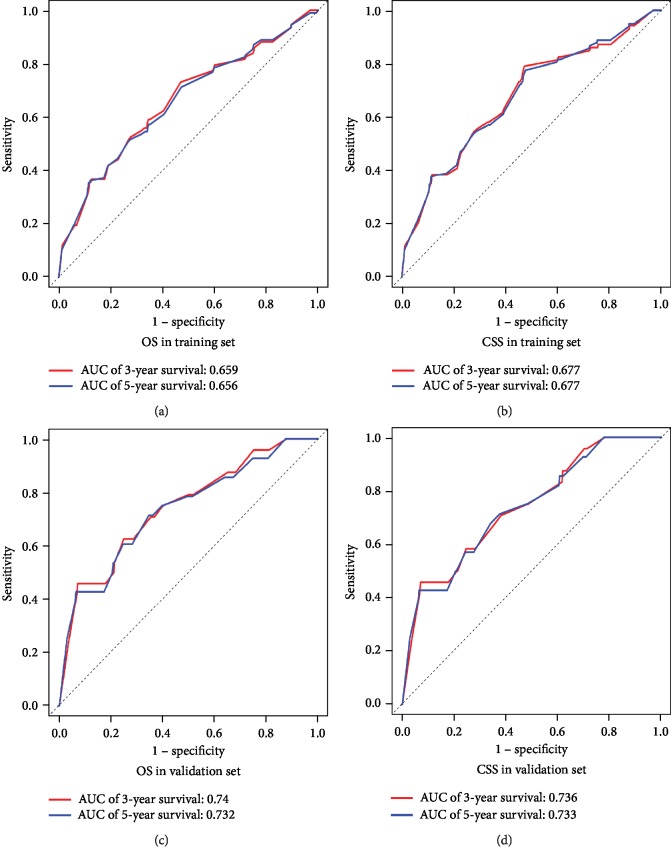
Receiver operating characteristic curves (ROC) of overall survival (a) and cancer-specific survival (b) in the training cohort; ROC of overall survival (c) and cancer-specific survival (d) in the validation cohort. Area under the receiver operating characteristic curve (AUC) values reflect the performance of the nomograms.

**Figure 4:**
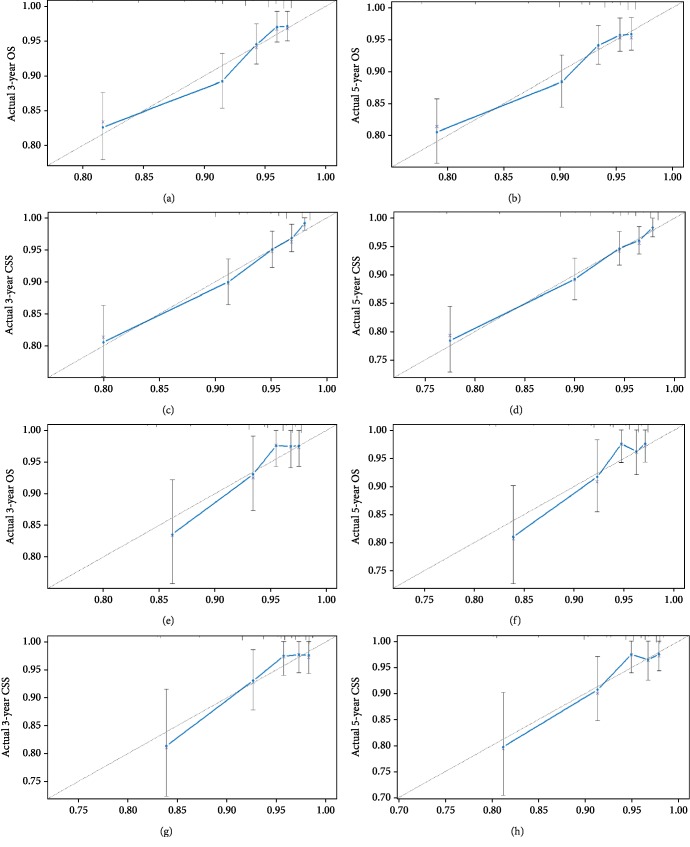
Internal calibration plots of 3-year (a) and 5-year (b) overall survival, and external calibration plots of 3-year (c) and 5-year (d) overall survival. Internal calibration plots for 3-year (e) and 5-year (f) cancer-specific survival, and external calibration plots for 3-year (g) and 5-year (h) cancer-specific survival. The cohort was equally divided into five groups to perform internal and external validation. *x*-axis and *y*-axis represent nomogram-predicted survival and actual survival, respectively. The dashed line stands for excellent agreement and closer distances between points, and the dashed line demonstrated better predicting ability.

In the training cohort, we calculated concordance indices (C-indices) for internal validation, which showed that C-indices for OS and CSS predictions were 0.699 (95% CI 0.652-0.746) and 0.734 (95% CI 0.690-0.778), respectively. In the validation cohort, C-indices for external validation were 0.704 (95% CI, 0.615-0.793) and 0.724 (95% CI, 0.636-0.812), respectively. The prognostic nomograms were validated both internally and externally. For the training cohort, area under the receiver operating characteristic curves (AUCs) were 0.659 and 0.656 for 3- and 5-year OS, and 0.677 for both 3- and 5-year CSS. Similarly, AUCs of 3- and 5-year OS in the validation cohort were 0.74 and 0.732, and those for 3- and 5-year CSS was calculated as 0.736 and 0.733, respectively. ROC curves demonstrated the satisfactory discriminative performance of our nomograms (Figure 3). Internal and external calibration plots indicated superior agreement between nomogram prediction and actual prognosis (Figure 4).

## 4. Discussion

It is universally acknowledged that diverse factors affect tumor development and patients' prognosis. Most previous studies focused on a single aspect of the prognosis of children with WT. Undoubtedly, judging a patient's prognosis through just a single variable may contribute to deviation. To deal with this issue, we integrated multiple prognostic factors to establish nomograms to predict 3- and 5-year OS and CSS of children with WT. Nomograms are critical components for decision-making in clinical practice because well-constructed nomograms provide accurate and personalized prognosis and aid clinicians to take the best therapeutic strategies. [21] Nomograms have been applied to predict many tumors, such as breast cancer, colorectal cancer, prostate cancer, endometrial cancer, osteosarcoma, and chondrosarcoma [16, 18, 22–25]. Nevertheless, we did not find prognostic nomograms for children with WT, so we were committed to develop such nomograms.

To maximize accuracy, we performed univariate and multivariate Cox's regression analyses and controlled for confounding variables while identifying prognostic factors. Five predictors, including age, tumor laterality, size, stage, and surgery were proven to be independent predictors for the survival of children with WT, whereas patients' gender and race were not significant prognostic factors. Different previous studies categorized patients as different age groups, and there was not a widely accepted classification, which might bring in different statistical analysis results. To solve this issue, for the first time, we used X-tile to determine the optimal cut-point of WT patients' age at diagnosis as 3 years old based on status and survival time. Pritchard-Jones et al. reported that an older age could be viewed as a prognostic factor attributing to poorer prognosis in stage I, favorable-histology WT, and the 4-year event-free survival (EFS) rate of children less than 2 years old, 2-4 years old, and 4 years old and older at diagnosis was 93.2%, 87.2% and 71.3%, respectively [10]. D'Angelo et al. also reported that children under 2 years old at diagnosis had better prognosis [1]. Other scholars also drew the conclusion that age at diagnosis was correlated with patients' prognosis, and this factor could determine the risk stratification and therapy [7, 8, 12, 26]. Our present study was in line with the above studies. Our statistical analyses and nomograms demonstrated that increasing age at diagnosis was an adverse independent factor for children with WT. Shamberger et al. reported that older age was associated with increased risk of local recurrence [27]. However, Vahudin et al. and Aronson et al. reported that age was not a significant prognostic factor [28, 29]. The possible reason might be the limitation of their small sample size which contained merely 65 and 57 patients, respectively. In the present study, we included a total of 1613 children in the SEER database, so our result might be more reliable.

Bilateral disease accounts for approximately five percent of WT. [30] Prior studies showed that bilateral WT was a challenge and had a worse prognosis [2]. Our nomograms showed higher risk scores in bilateral WT, which was consistent with previous studies. The most challenging issue was to completely resect bilateral tumors yet maintaining adequate nephrons to prevent renal failure [2, 31]. Cozzi et al. [32] reported that nephron-sparing surgery increased the risk of blood pressure hypertension, renal dysfunction possibility, cardiovascular disease, overall mortality, and end-stage renal disease, which are attributed to adverse outcomes. As for tumor size, our study demonstrated that it was an independent prognostic factor and the risk increased as the tumor became larger. Other scholars drew a similar conclusion, and it was estimated that the risk of death increased by 2% if the tumor volume after preoperative chemotherapy increased by 10 ml [33].

In terms of tumor stage, we confirmed it as a prognostic factor and found that distant tumor had the highest risk scores, followed by regional tumor. It was reported that tumor stage was determined as a critical prognostic factor for a long time [2, 26]. Distant tumor was associated with tumor metastasis and the most frequent distant site for WT metastases were pulmonary metastases; liver metastases were less common [2, 34]. Moreover, Varan reported that lungs and liver were two of the most frequent recurrence sites. Hence, distant metastatic tumors had higher risk for survival. Surgery was generally acknowledged as the most critical part of the therapy of WT. Several groups concluded that surgery played a paramount part in the therapy of WT. [2, 11, 35] The present study also identified surgery as a significant prognostic factor for children with WT. Our nomograms indicated that children who did not undergo surgery had a higher risk for 3- and 5-year OS and CSS survival, which again confirmed the significance of the surgery.

Utilizing a statistical analysis method, we identified independent prognostic factors and constructed prognostic nomograms by incorporating these prognostic factors to predict 3- and 5-year OS and CSS for children with WT. Previous studies indicated that chemotherapy and radiotherapy caused long-term side effects and were harmful to children's growth and development [11]. Consequently, it was of significance to evaluate the extent of children's risk and perform risk-based therapy. The nomograms could aid clinicians to precisely judge patients' conditions with merely basic clinical features.

Though the prognostic nomograms provided a relatively comprehensive forecast for children with WT, several limitations should be taken into consideration. First, some clinical laboratory results and other prognostic factors might also affect the survival of patients, such as proalbumin, blood sugar, lymph node involvement [28], and loss of heterozygosity at chromosomes 1p and 16q. [7] Chemotherapy and radiotherapy were not included in the present study due to the absence of relevant data in the SEER database, which might lead to incomprehension of prediction. Second, we did not stratify WT as more detailed subtypes such as favorable and unfavorable WT, which might limit the precision of prediction. Third, all the data we analyzed were collected from the SEER database. This might result in some bias. A validation cohort using another independent dataset for external validation could improve the credibility of the study. Despite some defects, these nomograms provided individualized and precise prediction of 3- and 5-year OS and CSS for children with WT.

## 5. Conclusion

Five prognostic factors, including children's age, tumor laterality, tumor size, use of surgery, and tumor stage, were confirmed to be independent prognostic factors for OS and CSS of children with WT. These independent prognostic variables were incorporated to establish nomograms which provided precise and convenient prediction of OS and CSS for children with WT. They were powerful tools which assisted clinicians to estimate personalized risk and execute optimal therapy.

## Figures and Tables

**Table 1:** Baseline clinicopathological features of patients with Wilms' tumor.

Variables	Training cohort (*n* = 1210)		Validation cohort (*n* = 403)		Total (*n* = 1613)		*P*
Sex (*n*, %)							0.480
Male	586	48.4%	187	46.4%	773	47.9%	
Female	624	51.6%	216	54.6%	840	52.1%	
Age (*n*, %)							0.967
≤3	755	62.4%	251	62.3%	1006	62.4%	
3-18	455	37.6%	152	37.7%	607	37.6%	
Race (*n*, %)							0.591
Black	215	17.8%	71	17.6%	286	17.7%	
White	925	76.4%	314	77.9%	1239	76.8%	
Other	70	5.8%	18	4.5%	88	5.5%	
Laterality (*n*, %)							0.621
Left	566	46.8%	188	46.7%	754	46.7%	
Right	573	47.3%	186	46.1%	759	47.1%	
Bilateral	71	5.9%	29	7.2%	100	6.2%	
Tumor size (*n*, %)							0.791
≤4	98	8.1%	37	9.2%	135	8.4%	
4-7	150	12.4%	50	12.4%	200	12.4%	
>7	962	79.5%	316	78.4%	1278	79.2%	
Surgical status (*n*, %)							0.085
No	29	2.4%	4	1%	33	2.0%	
Yes	1181	97.6%	399	99%	1580	98.0%	
SEER historic stage A							0.824
Regional	380	31.4%	123	30.5%	503	31.2%	
Localized	543	44.9%	188	46.7%	731	45.3%	
Distant	287	23.7%	92	22.8%	379	23.5%	

**Table 2:** Univariate Cox's regression analysis for OS and CSS in Wilms' tumor patients from the training cohort.

Variables	Overall survival			Cancer-specific survival		
	HR	95% CI	*P*	HR	95% CI	*P*
Sex						
Male	Reference			Reference		
Female	0.972	0.685-1.379	0.874	1.031	0.709-1.498	0.875
Age						
≤3	Reference			Reference		
3-18	1.917	1.351-2.721	<0.001	2.446	1.672-3.577	<0.001
Race						
Other	Reference			Reference		
White	0.837	0.406-1.723	0.629	0.737	0.356-1.524	0.411
Black	0.914	0.410-2.035	0.825	0.822	0.364-1.856	0.637
Laterality						
Left	Reference			Reference		
Right	0.778	0.534-1.134	0.191	0.740	0.493-1.110	0.145
Bilateral	2.370	1.366-4.111	0.002	2.486	1.403-4.405	0.002
Tumor size						
≤4	Reference			Reference		
4-7	2.703	1.014-7.204	0.047	3.333	0.965-11.512	0.057
>7	2.092	0.852-5.134	0.107	3.159	1.000-9.977	0.0499
Surgical status						
No	Reference			Reference		
Yes	0.258	0.131-0.509	<0.001	0.284	0.132-0.610	0.001
SEER historic stage A						
Regional	Reference			Reference		
Localized	0.704	0.433-1.145	0.158	0.482	0.279-0.832	0.009
Distant	2.716	1.770-4.167	<0.001	2.591	1.673-4.013	<0.001

**Table 3:** Multivariate Cox's regression analysis for OS and CSS in Wilms' tumor patients from the training cohort.

Variables	Overall survival			Cancer-specific survival		
	HR	95% CI	*P*	HR	95% CI	*P*
Age						
≤3	Reference			Reference		
3-18	1.745	1.206-2.524	0.003	2.155	1.442-3.220	<0.001
Laterality						
Left	Reference			Reference		
Right	0.783	0.538-1.141	0.203	0.747	0.497-1.121	0.158
Bilateral	1.887	1.036-3.435	0.038	2.263	1.221-4.195	0.009
Tumor size						
≤4	Reference			Reference		
4-7	2.991	1.117-8.010	0.029	3.622	1.044-12.566	0.043
>7	1.832	0.733-4.581	0.195	2.534	0.790-8.131	0.118
Surgical status						
No	Reference			Reference		
Yes	0.376	0.183-0.771	0.008	0.405	0.182-0.901	0.027
SEER historic stage A						
Regional	Reference			Reference		
Localized	0.728	0.443-1.194	0.209	0.527	0.302-0.919	0.024
Distant	2.307	1.482-3.589	<0.001	2.134	1.358-3.353	0.001

**Table 4:** Detailed scores of prognostic factors in the OS and CSS nomograms.

Characteristic	OS nomogram	CSS nomogram
Age		
≤3	0	0
3-18	5	5.6
Laterality		
Left	2.2	2.1
Right	0	0
Bilateral	7.7	7.9
Tumor size		
≤4	0	0
4-7	0.3	1.1
>7	0.7	2.2
Surgical status		
No	8.6	6.6
Yes	0	0
SEER historic stage A		
Regional	2.7	4.6
Localized	0	0
Distant	10	10

## Data Availability

The datasets analyzed during the current study were downloaded from the Surveillance, Epidemiology, and End Results database (https://seer.cancer.gov/).

## Abbreviations

WT: Wilms' tumor
CSS: Cancer-specific survival
OS: Overall survival
SEER: Surveillance, Epidemiology, and End Results
C-indices: Concordance indices.
